# Тиреотропин-секретирующие аденомы гипофиза: клинические особенности и результаты лечения 45 пациентов

**DOI:** 10.14341/probl13325

**Published:** 2023-09-27

**Authors:** Д. А. Трухина, Е. Г. Пржиялковская, Ж. Е. Белая, А. Ю. Григорьев, В. Н. Азизян, Е. О. Мамедова, Л. Я. Рожинская, А. М. Лапшина, Е. А. Пигарова, Л. К. Дзеранова, Н. М. Платонова, Е. А. Трошина, Г. А. Мельниченко

**Affiliations:** Национальный медицинский исследовательский центр эндокринологии; Национальный медицинский исследовательский центр эндокринологии; Национальный медицинский исследовательский центр эндокринологии; Национальный медицинский исследовательский центр эндокринологии; Национальный медицинский исследовательский центр эндокринологии; Национальный медицинский исследовательский центр эндокринологии; Национальный медицинский исследовательский центр эндокринологии; Национальный медицинский исследовательский центр эндокринологии; Национальный медицинский исследовательский центр эндокринологии; Национальный медицинский исследовательский центр эндокринологии; Национальный медицинский исследовательский центр эндокринологии; Национальный медицинский исследовательский центр эндокринологии; Национальный медицинский исследовательский центр эндокринологии

**Keywords:** тиреотропин-секретирующие аденомы гипофиза, тиреотоксикоз, диагностика, лечение

## Abstract

**ОБОСНОВАНИЕ:**

ОБОСНОВАНИЕ. Тиреотропин-секретирующие аденомы гипофиза (ТТГ-АГ) являются редкой причиной тиреотоксикоза и составляют 0,5–2% от всех аденом гипофиза. Учитывая редкость заболевания, анализ каждого случая ТТГ-АГ является чрезвычайно актуальным.

**ЦЕЛЬ:**

ЦЕЛЬ. Провести анализ характеристик и результатов лечения пациентов с ТТГ-АГ, а также определить предоперационные и ранние послеоперационные факторы, предсказывающие длительную ремиссию заболевания.

**МАТЕРИАЛЫ И МЕТОДЫ:**

МАТЕРИАЛЫ И МЕТОДЫ. В одноцентровом ретроспективном исследовании проанализированы клинические признаки, лабораторные и инструментальные исследования, а также результаты лечения пациентов с ТТГ-АГ в период с 2010-го по 2023 гг. Предоперационные факторы, а также уровень ТТГ, измеренный на 3 сутки после операции, оценивали на предмет их способности предсказывать долгосрочную ремиссию при сравнении групп пациентов с ТТГ-АГ с ремиссией и без ремиссии.

**РЕЗУЛЬТАТЫ:**

РЕЗУЛЬТАТЫ. В исследование было включено 45 пациентов с ТТГ-АГ (14 мужчин, 31 женщина), с медианой возраста 45 лет [30; 57]. Наиболее часто из клинических проявлений ТТГ-АГ встречались: нарушения ритма сердца — у 37 (82,2%) пациентов, патология щитовидной железы — у 27 (60%), неврологические нарушения — у 24 (53,35%). Большинство АГ были представлены макроаденомами (n=35, 77,8%). До операции аналоги соматостатина получали 28 (77,8%) пациентов, и у 20 (71,4%) на момент проведения оперативного вмешательства был эутиреоз. Хирургическое лечение проведено 36 (80%) пациентам, послеоперационная ремиссия достигнута в 31 случае (86,1%). При назначении аналогов соматостатина пациентам без ремиссии/с рецидивом после операции ремиссия наступила в 100% случаев (4/4). Увеличение размера АГ на 1 мм повышает вероятность рецидива/отсутствия ремиссии в 1,15 раза, а инвазия АГ в ходе операции — в 5,129 раза. Уровень ТТГ на 3 сутки после оперативного вмешательства выше 0,391 мМЕ/л (AUC — 0,952; 95% ДИ 0,873-1,000; станд. ошибка 0,04; р<0,001) позволяет выделить группу риска рецидива/отсутствия ремиссии после хирургического лечения (чувствительность = 100%, специфичность = 88,9%).

**ЗАКЛЮЧЕНИЕ:**

ЗАКЛЮЧЕНИЕ. ТТГ-АГ в структуре АГ встречаются крайне редко, вследствие чего в большинстве своем неправильно диагностируются и выявляются уже на стадии макроаденомы. Наиболее эффективным методом лечения является трансназальная трансфеноидальная аденомэктомия. В качестве терапии второй линии при неэффективности оперативного лечения могут использоваться аналоги соматостатина. Нами предложена возможная модель оценки уровня послеоперационного ТТГ (>0,391 мЕд/л) для прогнозирования рецидивов ТТГ-АГ, что требует подтверждения на расширенной выборке пациентов.

## Abstract (en)

**BACKGROUND:**

BACKGROUND: Thyrotropin-secreting pituitary adenomas (TSH-PA) are a rare cause of thyrotoxicosis and account for 0.5-2% of all pituitary adenomas. Taking into account the rarity of the disease, it is extremely important to analyze each case of TSH-PA. AIM: To analyze the clinical characteristics and treatment outcomes of patients with TSH-PA, as well as to determine preoperative and early postoperative factors that predict long-term remission.

**MATERIALS AND METHODS:**

MATERIALS AND METHODS: In a single-center retrospective study we analyzed clinical signs, laboratory and instrumental studies, as well as the treatment outcomes of patients with TSH-PA from 2010 to 2023. Preoperative factors, as well as TSH level measured on day 3 postoperatively, were evaluated for their ability to predict long-term remission when comparing groups of patients with and without remission. RESULTS: The study included 45 patients with TSH-PA (14 men, 31 women), with a median age of 45 years [30; 57]. The most common clinical manifestations of TSH-PA were: cardiac arrhythmia in 37 (82.2%) patients, thyroid pathology in 27 (60%), neurological disorders in 24 (53.35%). Most PAs were macroadenomas (n=35, 77.8%). Preoperatively, 28 (77.8%) patients received somatostatin analogs, and 20 (71.4%) patients were euthyroid at the time of surgery. Surgical treatment was performed in 36 (80%) patients, postoperative remission was achieved in 31 cases (86.1%). Administration of somatostatin analogues to patients with no remission/relapse after surgery lead to the remission in 100% of cases (4/4). A 1 mm increase in PA size raised the odds of recurrence/no remission by 1.15-fold,and PA invasion during surgery — by  5.129 fold. A TSH level on day 3 postoperatively above 0.391 mIU/L (AUC, 0.952; 95% CI 0.873–1.000; standard error 0.04; p<0.001) identifies patients with relapse/absence of remission after surgical treatment (sensitivity = 100%, specificity = 88.9%).

**CONCLUSION:**

CONCLUSION: The TSH-PA in the structure of PAs is extremely rare, and as a result, most of them are misdiagnosed and detected already at the stage of macroadenoma. The most effective method of treatment is transnasal transsphenoidal adenomectomy. Somatostatin analogues can be used as second-line therapy if surgical treatment is ineffective. We have proposed a possible model for postoperative TSH levels (>0.391 mU/l) to predict recurrence of TSH-PA, which requires validation on an expanded number of cases.

## ВВЕДЕНИЕ

Аденомы гипофиза, секретирующие тиреотропный гормон (ТТГ-АГ), встречаются нечасто и составляют 0,5–2% от всех АГ, являясь также достаточно редкой причиной тиреотоксикоза [1]. Подавляющее большинство ТТГ-АГ — это доброкачественные опухоли, и в литературе описано лишь три ТТГ-продуцирующие карциномы [2]. Первый случай ТТГ-АГ был задокументирован Jailer J.W. и Holub D.A. в 1960 г., они предположили и доказали, что синдром тиреотоксикоза может быть связан с АГ [3]. Всего же в мировой литературе описано около 540 пациентов с ТТГ-АГ [4][5]; в российской литературе представлено 29 случаев [6][7][8]. Аденомы, секретирующие ТТГ, также могут входить в состав наследственных синдромов, таких как синдром множественных эндокринных неоплазий 1 типа (мутации в гене MEN1); кроме того, описано 2 случая с мутацией в гене AIP (синдром семейных изолированных АГ) [9][10]. В основном (~70%) ТТГ-АГ секретируют только ТТГ, остальные 30% приходятся на смешанные АГ, а самым частым сочетанием являются ко-секреция с соматотропным гормоном (СТГ) или пролактином (ПРЛ) [9].

Ранее ТТГ-АГ диагностировались на стадии инвазивной макроаденомы и считались трудноизлечимыми. Однако появление новых методов иммунометрических анализов для оценки функции щитовидной железы (ЩЖ), инструментальных методов диагностики с высокой разрешающей способностью значительно улучшили диагностику пациентов с синдромом тиреотоксикоза, что позволило чаще выявлять случаи центрального тиреотоксикоза [1]. Тиреотропиному следует подозревать при наличии неадекватного уровня ТТГ (нормального или повышенного) у лиц с повышенными уровнями тиреоидных гормонов. В начале диагностики центрального тиреотоксикоза всегда необходимо проведение повторного лабораторного тестирования для исключения лабораторных ошибок и преходящих изменений уровней гормонов ЩЖ. В дальнейшем для постановки диагноза ТТГ-АГ и дифференциальной диагностики с синдромом резистентности к тиреоидным гормонам могут применяться высокочувствительные функциональные пробы (проба с тиреотропин-рилизинг-гормоном, проба подавления трийодтиронином), определение молярного соотношения α-субъединицы/ТТГ, поиск мутаций в гене THRB [1][5][11]). Также в некоторых случаях для диагностики ТТГ-АГ возможна пробная терапия аналогами соматостатина (АС) длительного действия (в течение не менее двух месяцев) [9][12] и АС короткого действия [13]. Несмотря на все вышесказанное, согласно работе De Herdt С. и соавт., на момент диагностики ТТГ-АГ у пациентов в большей части случаев, по данным визуализирующих исследований, выявляются макроаденомы гипофиза, хотя и сообщается о неуклонном снижении их доли [5][14][15][16].

Длительный период до постановки верного диагноза может быть связан с неярко выраженной клинической картиной тиреотоксикоза, а при макроаденомах на первый план может выходить неврологическая симптоматика [1]. Исследование только уровня ТТГ в рамках исключения функциональных нарушений щитовидной железы также может приводить к ошибочной тактике ведения пациентов и «пропуску» случаев центрального тиреотоксикоза, ввиду часто встречающихся нормальных показателей ТТГ при повышенных уровнях свободных фракций тироксина (свТ4) и/или трийодтиронина (свТ3) [17]. В Российской Федерации постановка диагноза ТТГ-АГ в сложных ситуациях ограничена в связи с недоступностью тиреотропин-рилизинг-гормона, трийодтиронина [6].

Лечением первой линии ТТГ-АГ считается транссфеноидальная аденомэктомия. По данным метаанализа, биохимическая ремиссия после оперативного вмешательства составляет 69,7% (95% доверительный интервал (ДИ) 61,1–78,4%), и, хотя имеются исследования, где процент послеоперационной ремиссии равен 100%, ее уровень в целом не достигает желаемых результатов [4]. Введение АС перед оперативным вмешательством может способствовать уменьшению размера АГ в связи с экспрессией рецепторов соматостатина ТТГ-АГ [16]. Тем не менее до сих пор не существует единого мнения относительно необходимости неоадъювантной терапии АС ввиду отсутствия повышения процента радикального удаления опухоли, а также связи достижения в предоперационном периоде эутиреоидного статуса с результатами хирургического лечения [5]. При неэффективности оперативного вмешательства, отказе от него или невозможности проведения операции могут быть назначены АС для контроля заболевания: послеоперационное применение АС приводит к стабилизации заболевания в 81,9% случаев [5][16].

Применение агонистов дофаминовых рецепторов (АДР), особенно каберголина, эффективно в основном при АГ со смешанным типом секреции — ПРЛ+ТТГ. В качестве монотерапии ТТГ-АГ результаты лечения АДР противоречивы [1][18]. Назначение АС и АДР одновременно при наличии экспрессии соматостатиновых (SSTR) и дофаминовых рецепторов D2 (D2R) в удаленной опухолевой ткани в целом может быть эффективно для контроля заболевания при неуспешном оперативном вмешательстве [4].

В связи с высокой эффективностью АС, риском развития пангипопитуитаризма и других осложнений после лучевой терапии, этот метод лечения в настоящее время применяется значительно реже. Однако при неэффективности оперативного вмешательства, отсутствии эффекта от медикаментозного лечения, агрессивном росте опухоли возможно применение лучевой терапии с последующим назначением лекарственных препаратов [4][5][9].

В связи с редкостью заболевания и большой разнородностью применяемых методов диагностики ремиссии, критерии излечения пациентов с ТТГ-АГ четко не установлены. Неопределяемый уровень ТТГ через неделю после операции может свидетельствовать о полной резекции опухоли, при условии отсутствия применения иных методов лечения перед оперативным вмешательством [19]. В исследовании Kim S.H. и соавт. было выявлено, что уровень ТТГ через 12 часов после операции был самым сильным предиктором ремиссии с пороговым значением 0,62 мкМЕ/мл [20]. Нормализация молярного соотношения α-субъединицы/ТТГ в целом также является хорошим показателем для оценки эффективности лечения, однако он характеризуется небольшой чувствительностью, так как примерно у 25% пациентов с ТТГ-АГ данное соотношение находится в норме. Наиболее чувствительным и специфичным тестом для подтверждения полного удаления аденомы, при отсутствии противопоказаний, остается проба с Т3 [21], что требует поиска других методов диагностики длительной ремиссии у пациентов с ТТГ-АГ в условиях недоступности проведения функциональных тестов.

Данные, собранные о ТТГ-АГ, чрезвычайно актуальны, и каждый случай может способствовать расширению осведомленности об этом заболевании и увеличению клинического опыта в мировой практике. Цель настоящего исследования состояла в анализе клинических характеристик, особенностей диагностики пациентов при недоступности проведения функциональных проб, лечения, критериев ремиссии и последующего наблюдения 45 пациентов с ТТГ-АГ, обследованных в одном лечебном учреждении.

## МЕТОДЫ

## Дизайн исследования

Проведено одноцентровое обсервационное ретроспективное исследование.

## Критерии соответствия

Пациенты с АГ и нормальным или повышенным уровнем ТТГ в крови в сочетании с повышенными уровнями свТ4 и/или свТ3; пациенты с подтвержденным, по данным ИГХ, диагнозом ТТГ-АГ.

## Условия проведения

Набор пациентов проводился на базе ФГБУ «НМИЦ эндокринологии» Минздрава России с 2010 по 2023 гг.

Медицинские записи пациентов были проанализированы ретроспективно. Собранная информация включала демографические данные, анамнез заболевания, физикальное обследование, лабораторные и инструментальные исследования, методы лечения, гистологические результаты.

Диагноз ТТГ-АГ выставлялся на основании гормонального обследования (неадекватный уровень ТТГ при высоких значениях свТ4 и/или свТ3), клинической картины, данных магнитно-резонансной томографии (МРТ) гипофиза. Некоторым пациентам проводился тест с октреотидом длительного действия (20 мг внутримышечно 1 раз в 28 дней или ланреотид 120 мг подкожно 1 раз в 28 дней в течение не менее двух месяцев).

Определение уровней ТТГ, свТ4, свТ3 проводилось до и после оперативного вмешательства на Architect i2000SR (Abbott Laboratories, Abbott Park, Illinois, U.S.A, референсные интервалы: ТТГ (0,25-3,5 мМЕ/л), свТ4 (9–20 пмоль/л), свТ3 (2,5–5,5 пмоль/л).

Всем пациентам исключался смешанный характер секреции АГ: проводился забор крови на определение уровней гормонов передней доли гипофиза и их органов-мишеней (СТГ, ПРЛ, фолликулостимулирующий гормон (ФСГ), лютеинизирующий гормон (ЛГ), инсулиноподобный фактор роста 1 (ИФР-1), кортизол крови утром, эстрадиол, тестостерон). У некоторых пациентов при необходимости выполнялся пероральный глюкозотолератный тест с 75 г глюкозы с определением уровней СТГ (0, 30, 60, 90, 120 мин) для исключения/подтверждения акромегалии. Также для исключения тканевого тиреотоксикоза исследовались уровни С-концевого телопептида коллагена 1 типа (СТх), остеокальцина, глобулина, связывающего половые гормоны (ГСПГ), общего холестерина.

Инструментальные исследования включали проведение МРТ, УЗИ ЩЖ (для исключения увеличения ЩЖ/узловых образований), рентгеновской денситометрии поясничного отдела позвоночника, проксимального отдела бедренной кости, электрокардиографии, эхокардиографии.

В рамках предоперационной подготовки для снижения уровней тиреоидных гормонов проводились инъекции октреотида короткого действия.

Оперативное лечение ТТГ-АГ проведено 36 пациентам; из них 34 пациента оперированы в ФГБУ «НМИЦ эндокринологии» Минздрава России. Операционный материал окрашивали гематоксилином и эозином по стандартной методике. Кроме того, в большинстве случаев выполнялось иммуногистохимическое (ИГХ) исследование послеоперационного материала для подтверждения диагноза: антитела (АТ) к ТТГ, СТГ, ПРЛ, Ki-67, адренокортикотропному гормону (АКТГ), ФСГ, ЛГ, соматостатиновым рецепторам 2 подтипа, дофаминовым рецепторам 2 подтипа, САМ5.2. При отсутствии ИГХ-исследования диагноз ТТГ-АГ подтверждался достижением послеоперационной ремиссии.

Критериями послеоперационной, а также медикаментозной ремиссии считались нормализация уровней ТТГ, свТ4, свТ3, регресс симптомов тиреотоксикоза, отсутствие, по данным МРТ в динамике, остаточной опухолевой ткани.

## Этическая экспертиза

Протокол исследования одобрен на заседании локального этического комитета ФГБУ «НМИЦ эндокринологии» Минздрава России от 22.02.2023, протокол № 4.

## Статистический анализ

Размер выборки предварительно не рассчитывался. Для статистической обработки материала использовались программы Statistica 13.3 (StatSoft США), SPSS 23 (Armonk, NY, IBM Corp). Для анализа распределения выборки использованы критерии Shapiro-Wilk и Kolmogorov-Smirnov. Данные описательной статистики представлены в виде медианы, а также 25-го и 75-го перцентилей. Для описания качественных данных рассчитывали абсолютные (n) и относительные значения (%). Для межгрупповых сравнений по количественным признакам применен критерий Mann-Whitney. Для анализа связей между категориальными переменными использовали критерий χ-квадрат Пирсона и точный критерий Фишера. Для оценки наличия взаимосвязи между признаками применен метод ранговой корреляции по Spearman. Для прогнозирования вероятности возникновения события выполнена логистическая регрессия. Статистически значимыми считали различия при p<0,05. Для устранения эффекта множественных сравнений применялась поправка Бонферрони.

## РЕЗУЛЬТАТЫ

## Характеристика пациентов

По данным регистра опухолей гипоталамо-гипофизарной области (ОГГО) из 85 регионов Российской Федерации на 29 марта 2023 г. выявлено 11 754 пациента с АГ, из них ТТГ-АГ (за исключением случаев, представленных в данной статье) составили 0,07% (n=8). В наше одноцентровое исследование включено 45 пациентов с ТТГ-АГ. Медиана (Me) возраста составила 45 лет [ 30; 57]; соотношение мужчин и женщин составило 1:2,2 (14 мужчин). Me лет до установки диагноза ТТГ-АГ у пациентов составила 3 [ 2; 6] года, максимально — 31 год.

Наиболее часто из клинических проявлений ТТГ-АГ встречались: нарушения ритма сердца (НРС) — 82,2% (n=37) (самым частым НРС была синусовая тахикардия (в 30 случаях)); неврологические нарушения (головная боль) — 53,3%; снижение МПК — в 46,7% случаев (остеопороз — 22,2%, у четырех пациентов имелись низкотравматичные переломы). Снижение массы тела наблюдалось всего в 22,2% случаев (ИМТ — 24,4 кг/м² [ 20,9; 28,3]). У 23,8% женщин репродуктивного возраста (n=21) выявлено нарушение менструального цикла. У одного пациента из симптомов были только неврологические нарушения вследствие макроаденомы гипофиза, и диагноз ТТГ-АГ был установлен лишь после проведения ИГХ. Патология ЩЖ обнаружена у 27/45 пациентов (60%): в большинстве случаев выявлены узловые образования ЩЖ (22,2%, n=10); Ме объема ЩЖ составила 16 мл [ 11,4; 26,6]. Все клинические проявления ТТГ-АГ представлены в таблице 1. Оперативное вмешательство на ЩЖ проводилось 1 пациенту до установки диагноза ТТГ-АГ — субтотальная тиреоидэктомия по поводу неверно установленного диагноза диффузного токсического зоба, при гистологическом исследовании операционного материала выявлен микрофокус папиллярного рака фолликулярного типа строения Т1N0Mх. Тиреостатики в анамнезе получали 26/45 пациентов (57,7%).

**Table:** Таблица 1. Клинические проявления ТТГ-АГ Table 1. Clinical manifestations of TSH-AH

Параметры	Значение
Нарушения ритма сердца, n (%)	37/45 (82,2)
- синусовая тахикардия - фибрилляция предсердий - желудочковая экстрасистолия	- 30 (66,7) - 6 (13,4) - 1 (2,2)
Патология щитовидной железы, n (%)	27/45 (60)
- диффузное увеличение - узловые образования - оба параметра	- 7 (15,6) - 10(22,2) - 10 (22,2)
Неврологические нарушения (головная боль), n (%)	24/45 (53,3)
Снижение МПК	21/45
- остеопороз - остеопения	- 10 (22,2) - 11 (24,4)
Тремор, n (%)	16/45 (35,6)
Нарушение менструального цикла, n (%)	5/21 (23,8)
Снижение массы тела, n (%)	10/45 (22,2)
Нарушение зрения, n (%)	4/45 (8,9)
Семейный анамнез, n (%)	0/45 (0)

## Лабораторные показатели

При первичном обследовании у 21 пациента выявлено повышение уровней ТТГ, свТ3 и свТ4; у 10 пациентов — повышение уровней свТ3 и свТ4 при нормальном ТТГ; у 7 пациентов повышение ТТГ и либо свТ3, либо свТ4; у 5 — один из гормонов (рис. 1–3). Медиана ТТГ составила 4,41 мМЕ/л [ 3,02; 6,65], свТ3 — 7,62 пмоль/л [ 6,2; 10,5], свТ4 — 22,6 пмоль/л [ 19,48; 29,35]. Также стоит отметить, что ТТГ был незначительно выше у пациентов, получавших тиреостатики, чем у тех, кто их не принимал.

**Figure:**
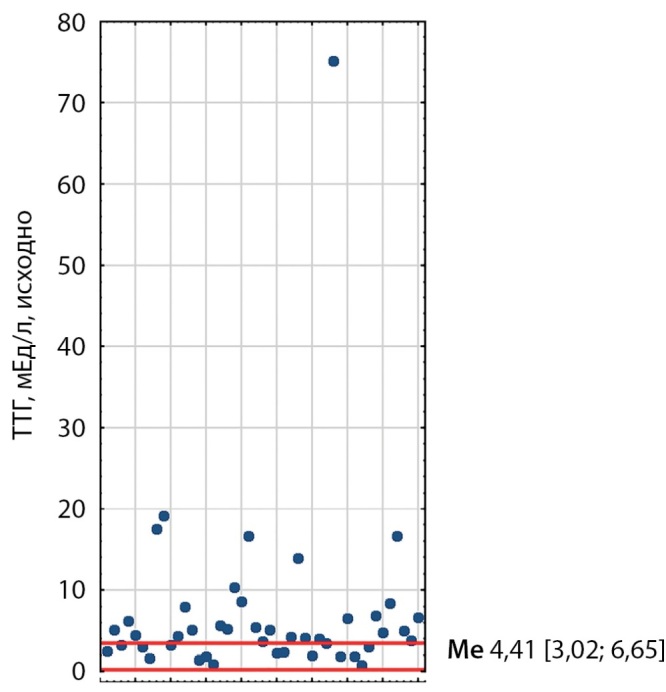
Рисунок 1. Распределение исходных показателей ТТГ у пациентов с ТТГ-АГ. Красными линиями обозначены референсные интервалы (0,25–3,5). Me [ Q1; Q3] — медиана, интерквартильный размах. Figure 1. Distribution of baseline TSH values in patients with TSH-AH. Red lines indicate reference intervals (0.25–3.5). Me [ Q1; Q3] — median, interquartile range

**Figure:**
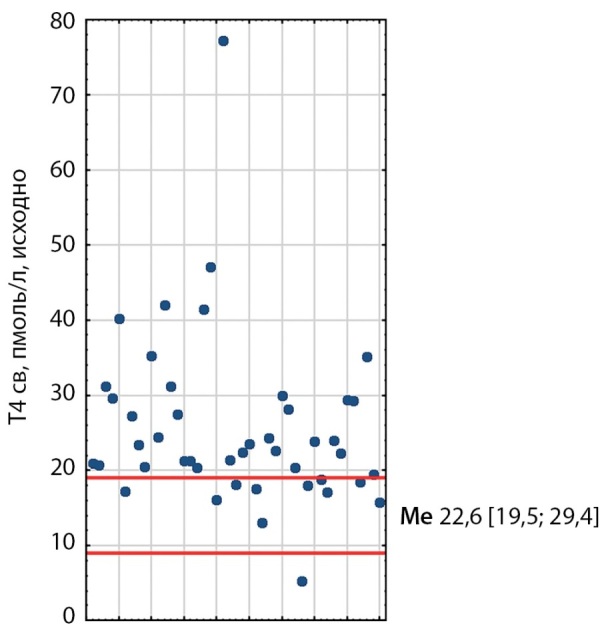
Рисунок 2. Распределение исходных показателей свТ4 у пациентов с ТТГ-АГ. Красными линиями обозначены референсные интервалы (9–19). Me [ Q1; Q3] — медиана, интерквартильный размах. Figure 2. Distribution of baseline fT4 values in patients with TSH-AH. Red lines indicate reference intervals (9–19). Me [ Q1; Q3]—median, interquartile range.

**Figure:**
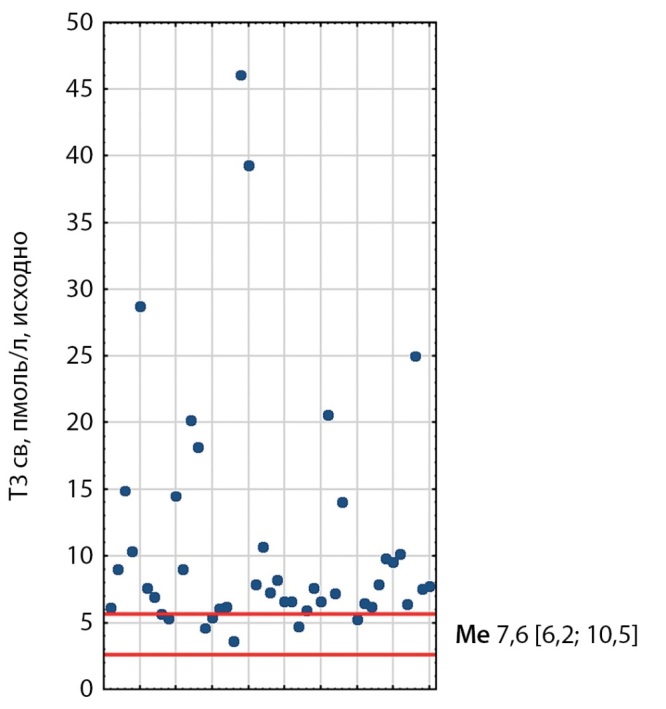
Рисунок 3. Распределение исходных показателей свТ3 у пациентов с ТТГ-АГ. Красными линиями обозначены референсные интервалы (2,6–5,7). Me [ Q1; Q3] — медиана, интерквартильный размах. Figure 3. Distribution of baseline fT3 values in patients with TSH-AH. Red lines indicate reference intervals (2.6–5.7). Me [ Q1; Q3]—median, interquartile range.

Из пациентов с повышением одного показателя:

Пациенту с отсутствием симптомов тиреотоксикоза (только неврологическая симптоматика) не выполнялось исследование свТ3, а значения ТТГ и свТ4 были в референсном интервале.

Повышение уровней СССГ, С-концевого телопептида коллагена 1-го типа, остеокальцина было выявлено в 60,53, 54,55 и 39,29% случаев соответственно. Уровни общего холестерина (ХС), липопротеидов низкой плотности, щелочной фосфатазы, антител к рецептору ТТГ не имели клинически значимых отклонений. В 8 случаях выявлена сочетанная секреция СТГ/ТТГ; гиперпролактинемия из-за масс-эффекта установлена в 7 случаях. Сочетание секреции ТТГ/АКТГ в нашей когорте пациентов не обнаружено.

При проведении корреляционного анализа не выявлено корреляций между уровнем ТТГ и свТ4/свТ3 (p>0,05), также не выявлено корреляций между уровнем ТТГ/свТ4/свТ3 и максимальным размером АГ (p>0,05).

## Проба с аналогами соматостатина

Проба с аналогами соматостатина проведена у 39/45 пациентов. Тест с октреотидом короткого действия проводился у 26 пациентов, из них у 15 произошла полная нормализация гормонов ЩЖ, у 6 снизились уровни ТТГ и свТ3 до нормальных значений, а свТ4 приблизился к верхней границе нормы.

Проба с октреотидом длительного действия была выполнена у 13/39 пациентов: у 7 пациентов достигнута полная нормализация гормонов ЩЖ; у 1 пациента выявлена нормализация уровней ТТГ, а значения свТ4 и/или свТ3 приблизились к верхней границе нормы. У 1 пациента с СТГ/ТТГ-АГ нормализовались гормоны ЩЖ, но не ИФР-1. Октреотид в дозе 20 мг 1 раз в 28 дней получали 11 пациентов, ланреотид в дозе 120 мг — 2 пациента.

У 8 пациентов отмечены побочные эффекты после применения аналогов соматостатина: диарея в 6 случаях, боли в животе — в 5, а также зарегистрированы тошнота, рвота, метеоризм, стеаторея, потеря массы тела, формирование камня в желчном пузыре менее, чем за год.

## Магнитно-резонансная томография

Большинство АГ представлены макроаденомами (77,8%), с Me диаметра опухоли 15,5 мм [ 12; 26] (табл. 2). Экстраселлярное распространение опухоли обнаружено в 21 случае.

**Table:** Таблица 2. Результаты МРТ у пациентов с ТТГ-АГ Table 2. MRI results in patients with TSH-AH

Характеристика АГ, n (%)	
- макроаденома	35 (77,8)
макс. диаметр опухоли (мм), Me [ Q1; Q3]	15,5 [ 12; 26]
экстраселлярный рост	21 (60)
- микроаденома	10 (22,2)
макс. диаметр опухоли (мм), Me [ Q1; Q3]	5 [ 4; 6]

## Результаты лечения

До операции с целью достижения эутиреоза, уменьшения клинической симптоматики октреотид получали 28/36 пациентов: 6 — октреотид длительного действия, 22 — короткого. Ме применения АС перед проведением оперативного вмешательства короткого действия составила 1 неделю [ 0,5; 2,2], длительного действия — 12 недель [ 8; 29]. Октреотид длительного действия в дозе 20 мг 1 раз в 28 дней принимали 5 пациентов, ланреотид 120 мг — 1 пациент.

Оперативное вмешательство проведено 36/45 пациентам (из них макроАГ — 29, микроАГ — 7). После первичной операции ранней послеоперационной ремиссии (РПР) достиг 31 пациент (86,1%). Медиана ТТГ после операции составила 0,161 мМЕ/л [ 0,016; 0,479], свТ3 — 3,06 пмоль/л [ 2,77; 3,73], свТ4 — 14,21 пмоль/л [ 10,75; 16,63]. У 15 пациентов уровень ТТГ был менее 0,1 мМЕ/л, у 15 — 0,1–1,0 мМЕ/л, у 4 — 1,0–3,7 мМЕ/л.

Инвазия опухоли в ходе операции выявлена в 12 случаях (макроАГ — 10 случаев; микроАГ — 2). ИГХ-исследование послеоперационного материала выполнено 24 пациентам. В 23 случаях АГ выявлена экспрессия ТТГ, в одном случае — единичные клетки, экспрессирующие ТТГ. Экспрессия соматостатиновых рецепторов 2 типа (SSTR2) выявлена во всех 15 случаях, в которых проводилось данное исследование; также выявлена экспрессия дофаминовых рецепторов D2 (D2R) во всех 10 случаях, где исследовался данный признак. ИГХ Ki-67 выполнено на 18 образцах АГ: у 4 образцов Ki-67 было менее 1%, у трех — от 1 до 3%, у четырех — более 3% (Me=4 [ 1,8; 10,4]).

После оперативного вмешательства у 5 пациентов диагностировано отсутствие ремиссии заболевания, у 3 пациентов — рецидив (табл. 3). Повторной операции подверглись 2 пациента (1 пациент с отсутствием ремиссии и 1 пациент с рецидивом), у обоих после операции достигнута ремиссия.

**Table:** Таблица 3. Сводная таблица с результатами лечения пациентов с ТТГ-АГ Table 3. Summary table with treatment results for patients with TSH-AH

Метод лечения	Количество пациентов
n, (%)	Ремиссия	Отсутствие ремиссии/рецидив
Оперативное вмешательство	36/45 (80)	31	5/3
Повторное оперативное вмешательство	2/8 (25)	2	–
АС длительного действия до оперативного вмешательства	6/36 (13,9)	4	2/–
Лучевая терапия после оперативного вмешательства	2/36 (5,6)	0	2/–
АС длительного действия после оперативного вмешательства	4/8 (50)	4	–
АС длительного действия после лучевой терапии	2/2 (100)	2	–
Каберголин	9/45 (20)	–	9/–
АС длительного действия (пациенты без оперативного вмешательства)	9/45 (20)	3	6/–

Лучевая терапия выполнена 2 пациентам после хирургического вмешательства и в обоих случаях ремиссия заболевания достигнута только последующим назначением АС длительного действия.

Терапия АС длительного действия после неуспешного хирургического лечения назначена 4 пациентам, ремиссия достигнута во всех случаях приема АС в дозах 10, 20, 30 мг 1 раз в 28 дней; продолжительность ремиссии у троих пациентов не менее двух лет. 9 пациентов получали агонисты дофаминовых рецепторов, но ни в одном случае монотерапии АДР эутиреоза не было достигнуто.

До оперативного вмешательства гипопитуитаризм выявлен у 4/34 пациентов (9,8%), после — у 10/34 (32,3%). У всех четырех пациентов до операции был вторичный гипогонадизм, у двух — вторичный гипокортицизм (2-НН).

Послеоперационные осложнения развились у 11 (34,4%) пациентов: у 4 пациентов — пангипопитуитаризм; у 2 — синдром неадекватной секреции АДГ; у 3 — транзиторный несахарный диабет; у 1 — транзиторный несахарный диабет и вторичная надпочечниковая недостаточность (2-НН); у 1 пациента развился СТГ-дефицит. Один пациент как до, так и после операции имел 2-НН и вторичный гипогонадизм. Два пациента по настоящее время находятся на заместительной терапии левотироксином натрия вследствие вторичного гипотиреоза.

Медиана наблюдения за прооперированными пациентами составила 12 мес. [ 1,5; 24]. Два пациента с РПР пропали из-под наблюдения. На выборке пациентов с ремиссией заболевания после как минимум 6 месяцев наблюдения рассчитана точка cut off послеоперационного ТТГ (на третьи сутки после операции) с целью выявить пациентов с риском рецидива заболевания при помощи ROC-анализа. Пациенты, которые не достигли ремиссии заболевания, и те, у которых в дальнейшем развился рецидив, имели уровень ТТГ после операции выше 0,391 мМЕ/л по критерию Юдена (AUC — 0,952; 95% ДИ 0,873–1,000; станд. ошибка – 0,04; р<0,001), специфичность (SP) = 88,9%, чувствительности (SE) = 100%.

Из 9 непрооперированных пациентов 9 назначены АС длительного действия. Хирургическое вмешательство не было произведено либо ввиду противопоказаний к его проведению, либо из-за отказа пациента. Только у 3/9 пациентов на момент окончания наблюдения достигнута ремиссия заболевания на фоне терапии АС (20 и 30 мг 1 раз в 28 дней). У одного из этих пациентов, по данным МРТ, выявлена макроАГ, у двух других — микроАГ; за время лечения аналогами соматостатина длительного действия отмечено уменьшение объема АГ у всех пациентов, в большей степени макроАГ (начальные размеры — 18х23х20 мм; размеры от последнего обследования — 12х18х15 мм).

Всего на момент окончания наблюдения из непрооперированных пациентов не достигли ремиссии 6 человек; после проведенного оперативного вмешательства — 1/36 человек (потерян из-под наблюдения).

4/6 пациента, не достигших ремиссии, находятся на терапии АС длительного действия; в настоящее время продолжается увеличение дозы АС для достижения контроля заболевания.

Одна непрооперированная пациентка с тяжелыми проявлениями тиреотоксикоза, с гигантской ТТГ-АГ умерла, не достигнув ремиссии, вследствие окклюзионной гидроцефалии. Также смерть была зарегистрирована у пациентки с ремиссией после повторного оперативного вмешательства — развилось психическое расстройство на фоне неконтролируемого приема десмопрессина по поводу послеоперационного несахарного диабета, не регрессировавшее после стабилизации тиреоидного статуса, нормализации уровня натрия; дестабилизация психического состояния привела к ступору, на фоне которого развилась пневмония, ставшая причиной смерти.

## Предоперационные факторы, влияющие на исход хирургического лечения

Для определения предоперационных факторов, влияющих на исход хирургического лечения, пациенты были разделены на 2 группы: долгосрочная ремиссия (n=18) и рецидив/отсутствие ремиссии (n=6). Критериями ремиссии считались отсутствие лабораторного тиреотоксикоза и остаточной ткани АГ по данным МРТ. Пациенты с РПР не были включены в анализ, минимальное время наблюдения за пациентом составило 6 мес., максимальное — 3 года.

По результатам сравнительной характеристики групп с ремиссией и отсутствием ремиссии/рецидивом статистически значимые различия по предоперационным уровням ТТГ, свТ3, свТ4, нарушению зрения, максимальному размеру опухоли, инвазии опухоли не были выявлены. Несмотря на отсутствие различий в вышеуказанных параметрах, отобрано 2 признака для проведения логистического регрессионного анализа с целью определения предоперационных факторов, влияющих на результат хирургического лечения: «инвазия АГ в ходе оперативного вмешательства» и «максимальный размер АГ».

Анализ показал, что увеличение размера АГ на 1 мм повышает вероятность рецидива/отсутствия ремиссии в 1,15 раза (ОШ 1,15, 95% ДИ 0,995–1,346, р=0,058); в группе отсутствия ремиссии/рецидива шанс инвазии АГ в ходе операции в 5,129 раза выше, чем в группе ремиссии (ОШ= 5,129, 95% ДИ 0,284–92,648; р=0,268). Учитывая тенденцию критерия p, скорее всего, увеличение числа пациентов в обеих группах может привести к усилению значимости результатов.

## ОБСУЖДЕНИЕ

В настоящей работе приведены клинические особенности и результаты лечения 45 пациентов с ТТГ-АГ. По данным регистра ОГГО РФ, редкие ТТГ-АГ составляют всего 0,45% от общего количества АГ. В мировой литературе на долю ТТГ-АГ также приходится от 0,5 до 2% от всех АГ [1], а распространенность в общей популяции составляет 1–2 случая на миллион человек, хотя, по данным шведского регистра, в последнее время она увеличилась до 2,8 на миллион человек [15]. Рост числа зарегистрированных случаев, скорее всего, связан с улучшением диагностических методов (лабораторных, инструментальных), а также с осведомленностью врачей об этом заболевании [22–24].

Как и в нашем исследовании (n=35 (77,8%)), процент выявления макроаденом ТТГ-АГ в мире все еще высок, так, в систематическом обзоре De Herdt C. и соавт. большинство ТТГ-АГ (76,9%) были макроаденомами. Однако сообщаются данные о неуклонном росте микроаденом в последние годы: в исследовании Yamada S. и соавт. количество микроаденом было значительно выше в недавно диагностированных случаях, чем в более ранние годы (p=0,0274) [16]; а по результатам метаанализа Cossu G. и соавт., процент микроаденом значительно увеличился в статьях, опубликованных после 2000 г. (p=0,04) [4].

По данным литературы, ТТГ-АГ встречаются с одинаковой частотой у мужчин и женщин, в отличие от других более распространенных заболеваний, сопровождающихся тиреотоксикозом, где преобладает женский пол [11]; возраст же постановки диагноза может быть любым, однако у большинства пациентов приходится на пятое-шестое десятилетие жизни и составляет примерно 42–46 лет [1][4][5][16]. В нашем исследовании соотношение мужчин и женщин составило 1:2,2, что несколько разнится с данными мировой практики, однако Ме возраста равнялась 45 годам [ 30; 57], как и в других работах.

Приблизительно в 1/3 случаев ТТГ-АГ ошибочно диагностируется как болезнь Грейвса или функциональная автономия, и, соответственно, увеличивается время до постановки верного диагноза и назначения адекватного лечения [15]. Некоторые пациенты могут подвергаться неоправданным радиойодтерапии (РЙТ) ¹³¹I и тиреоидэктомии, что, в свою очередь, может способствовать увеличению частоты возникновения инвазивных макроаденом [1]. Тем не менее не стоит забывать о том, что может быть сочетание ТТГ-АГ с болезнью Грейвса [25], с первичным гипотиреозом [26], а также в литературе обсуждается роль длительно некомпенсированного первичного гипотиреоза в развитии вторичной гиперплазии/вторичной ТТГ-АГ [27][28]. В нашей когорте пациентов Ме лет до установки диагноза ТТГ-АГ составила 3 года [ 2; 6], максимально — 31 год; тиреостатики принимали 26/45 пациентов (57,7%), а оперативному вмешательству на ЩЖ подвергся 1 пациент (роста АГ после тиреоидэктомии, по данным МРТ, не выявлено). У одного пациента в нашем исследовании выявлено сочетание первичного гипотиреоза с ТТГ-АГ (подтверждено по ИГХ); после трансназальной транссфеноидальной аденомэктомии пациент находится в ремиссии в течение трех лет, продолжает получать заместительную гормональную терапию левотироксином натрия.

У пациентов с ТТГ-АГ признаки и симптомы тиреотоксикоза чаще всего слабо выражены или могут вовсе отсутствовать, по сравнению с пациентами с другими причинами тиреотоксикоза [1][5][9]. Кроме того, при смешанных СТГ/ТТГ-АГ симптомы акромегалии могут выходить на первый план, поэтому важно при наличии АГ исследовать все гипофизарные гормоны [1]. В нашей работе у многих пациентов клинические проявления ТТГ-АГ были в мягкой форме, а у 1 пациента не имелось никаких признаков тиреотоксикоза, а были отмечены только лишь симптомы масс-эффекта опухоли (головные боли). У 82,2% пациентов выявлены нарушения ритма сердца — в основном синусовая тахикардия (n=30), фибрилляция предсердий диагностирована в 6 случаях, что соответствует данным других исследований [5][9][29]. Большинство ТТГ-АГ на момент постановки диагноза являются макроаденомами, что объясняет присутствие симптомов масс-эффекта опухоли у 30–40% пациентов [1][30]. В нашей работе неврологические нарушения (головные боли) выявлены чуть больше, чем у половины пациентов (n=24), а зрительные нарушения у 4. У 8 пациентов выявлена ко-секреция СТГ, также у 7 пациентов — повышение пролактина; однако мы не выявили экспрессии ЛГ/ФСГ или АКТГ в нашей серии случаев. Гиперсекреция СТГ и/или ПРЛ, наиболее распространена и присутствует примерно у 30% пациентов с ТТГ-АГ, что, скорее всего, связано с общими факторами транскрипции, такими как Prop-1 и Pit-1 [31]. Намного реже встречается смешанная ТТГ/ФСГ или ЛГ-АГ, в то время как на сегодняшний день не было зарегистрировано ТТГ/АКТГ-АГ, вероятно, из-за отдаленного происхождения кортикотрофных и тиреотрофных линий [1].

Диффузное увеличение ЩЖ выявлено у 7/45 пациентов, узловые образования в 10/45 случаях, а их сочетание — у 10. Увеличение частоты развития рака ЩЖ у пациентов с ТТГ-АГ является спорным вопросом. Так, имеются предположения, что повышенный риск РЩЖ у пациентов с ТТГ-АГ может быть обусловлен стимулирующим эффектом гиперсекреции ТТГ на тиреоциты [32], или более высокая распространенность РЩЖ у пациентов с ТТГ-АГ может быть связана с частым использованием УЗИ в этой конкретной когорте пациентов [5]. В нашей серии случаев РЩЖ выявлен у 1 пациента, задолго до установки верного диагноза ТТГ-АГ.

Диагноз ТТГ-АГ можно предположить при высоком или неадекватно нормальном уровне ТТГ у пациента с повышенными значениями свТ4 и/или свТ3. Первым шагом в диагностике центрального гипертиреоза является повторное определение тиреоидного статуса для исключения влияния разных веществ на сами лабораторные наборы [33] и преходящих изменений уровней гормонов ЩЖ на фоне некоторых состояний, например приема лекарственных препаратов (эстрогены, амиодарон), беременности, острых психических и критических состояний, подострого тиреоидита, генетических причин, включая резистентность к гормонам ЩЖ, семейную дисальбуминемическую гипертироксинемию. К тому же ТТГ-АГ следует заподозрить у пациентов с первичным гипотиреозом разной этиологии, у которых не удается достичь нормализации уровня ТТГ на фоне увеличения дозы левотироксина натрия при уверенности в комплаентности пациента [29]. Кроме вышеуказанных причин, с ТТГ-АГ необходимо дифференцировать синдром резистентности к тиреоидным гормонам (СРТГ) ввиду схожести их клинических проявлений и лабораторных показателей [34][35]. СРТГ — это доминантно наследуемое заболевание, обусловленное мутациями в гене THRB (80–85% случаев), с распространенностью 1 случай на 40 тыс. населения. Пациенты с СРТГ могут быть бессимптомными (в основном) или иметь симптомы тиреотоксикоза (реже) в зависимости от рефрактерности периферических тканей к высоким уровням свТ4 и свТ3. Отличить ТТГ-АГ от СРТГ можно при: наличии родственника первой линии родства с подобными симптомами, нормальном уровне α-субъединицы, отсутствии симптомов масс-эффекта АГ, повышении ТТГ на пробе с тиролиберином, отсутствии реакции на введение АС и оценке других показателей периферической крови, в частности СССГ и СТх [1][5][9]. В работе Han R. и соавт. проводилась дифференциальная диагностика пациентов с ТТГ-АГ и пациентов с СРТГ при помощи АС короткого действия [13]. Как у пациентов с ТТГ-АГ, так и у пациентов с СРТГ наблюдалось снижение уровня ТТГ в начале теста с АС (после первой инъекции), однако коэффициент подавления ТТГ через 24 часа по сравнению с 2 и 0 часами был значительно выше у пациентов с ТТГ-АГ, чем с СРТГ (70,58±18,6% против 6,01±25,41%, p<0,0001, 79,83±12,79% против 51,16±13,62%, р<0,0001 соответственно). В нашем исследовании октреотид короткого действия вводился 26/39 пациентам, из которых у 15 произошла полная нормализация гормонов ЩЖ, а проба с октреотидом длительного действия выполнена у 13/39 пациентов, из которых у 7 достигнута полная нормализация гормонов ЩЖ. Побочные эффекты были отмечены у 20,5% пациентов, в основном со стороны ЖКТ. В целом в рамках проведения дифференциальной диагностики ТТГ-АГ и СРТГ можно уверенно использовать тест с АС длительного действия, вводимый в течение не менее двух месяцев, в то время как тест с АС короткого действия может иметь низкую чувствительность и в настоящее время в НМИЦ эндокринологии используется только лишь в рамках предоперационной подготовки с целью купирования тиреотоксикоза.

Исследование только уровня ТТГ при подозрениях на нарушение функции гормонов ЩЖ также может затруднять диагностику ТТГ-АГ и приводить к неправильной тактике ведения пациентов ввиду нередко нормальных показателей ТТГ при измененных уровнях свТ4 и/или свТ3 [16]. В нашей работе нормальный уровень ТТГ на момент диагностики заболевания был зарегистрирован у 16/45 пациентов (35,6%).

Как было указано выше, для постановки диагноза ТТГ-АГ могут применятся диагностические пробы, оценка уровня α-субъединицы. У пациентов с ТТГ-АГ повышение уровня α-субъединицы может наблюдаться в 70% случаев и чаще всего при макроаденомах, исходя из этого нормальный уровень α-субъединицы делает диагноз ТТГ-АГ менее вероятным, но не исключает его [9]. При проведении пробы с тиролиберином (в/в введение 200 мкг тиролиберина) у относительно здорового контроля и пациентов с СРТГ наблюдается повышение уровня ТТГ в отличие от пациентов с ТТГ-АГ — отсутствие изменения уровня ТТГ наблюдается в 85% случаев. Проба с трийодтиронином представляет собой назначение 80–100 мкг трийодтиронина в сутки в течение 8–10 дней с последующей оценкой ТТГ, при ТТГ-АГ в 100% случаев отсутствует подавление уровня ТТГ, в отличие от других состояний. Однако проведение этого теста противопоказано пожилым людям и лицам с сердечно-сосудистыми заболеваниями, поскольку может вызывать нарушения ритма сердца, а также недоступно для проведения во многих странах [1][9][6]. Проба с АС длительного действия также может быть рекомендована для диагностики ТТГ-АГ. Так, у большинства пациентов с ТТГ-АГ наблюдается значительное снижение или нормализация уровней ТТГ, свТ4, свТ3 после инъекции АС. Применение же АС в качестве неоадъювантной терапии перед операцией способствует уменьшению размеров АГ, однако, по данным некоторых исследований, статистически значимых различий по улучшению исходов хирургического лечения при сравнении пациентов с назначением октреотида и без него не было выявлено [4][16][22][36]. В Российской Федерации пробы с тиролиберином и трийодтиронином, измерение α-субъединицы не проводятся, в клинической практике используется тест с АС короткого или длительного действия. В нашем исследовании проба с АС короткого и длительного действия была проведена у 39/45 пациентов, из них у 22 пациентов отмечена полная нормализация гормонов; предоперационно АС продолжали получать 28/36 пациентов с Ме применения АС короткого действия 1 неделю, длительного действия — 12 недель. АС не использовались при микроаденомах, не сопровождающихся выраженным повышением тиреоидных гормонов и выраженными симптомами тиреотоксикоза.

МРТ гипофиза рекомендуется проводить только после лабораторного подтверждения центрального тиреотоксикоза, вызванного ТТГ-АГ. Визуализирующие исследования могут не только подтвердить наличие ТТГ-АГ, но и помочь понять расположение опухоли, ее отношение к окружающей ткани. Большинство ТТГ-АГ представляют собой опухоли размером более 10 мм [9][22], однако, как было сказано ранее, в последнее время диагностируются все чаще микроТТГ-АГ. Если по данным МРТ не выявлено опухоли в турецком седле, а лабораторные анализы указывают на ТТГ-АГ, следует искать ТТГ-эктопию [37]. По результатам нашего исследования, по данным МРТ выявлено больше макроаденом с Ме максимального диаметра опухоли: 15,3 мм [1 2; 27], у микроаденом — 5 мм [ 4; 6]. Как и в других работах [5][16], корреляций между уровнем ТТГ/свТ4/свТ3 и размерами АГ не отмечено.

Терапией первой линии у пациентов с ТТГ-АГ является трансназальная транссфеноидальная аденомэктомия с дальнейшей ИГХ послеоперационного материала. По данным исследований, в настоящее время примерно у 80% пациентов с ТТГ-АГ можно достичь ремиссии при помощи только хирургического лечения [5][9]. ТТГ-АГ, по сравнению с другими АГ, имеет тенденцию к более высокой степени микроинвазии и внутри- и периопухолевого фиброза, что может ухудшать исход хирургического вмешательства, вызывать периоперационные осложнения, такие как кровотечение, ликворея, несахарный диабет и гипопитуитаризм [9][22]. Yamada S. и соавт. в своем исследовании на большой группе пациентов с ТТГ-АГ впервые выявили предоперационные факторы, которые могут влиять на исход хирургического вмешательства. Наиболее значимыми предикторами нерадикальной операции у пациентов являлись степень инвазии в кавернозные синусы, а также максимальный диаметр АГ — чем больше был диаметр опухоли и более выражена степень инвазии, тем чаще выявлялось отсутствие ремиссии после операции. Уровни ТТГ и свТ4, возраст и пол не влияли на исход [16].

В нашем исследовании процент ремиссий после проведения первичного оперативного вмешательства так же, как и в мире, достаточно высок — 86,1% (31/36 пациентов). Инвазия опухоли в ходе оперативного вмешательства выявлена у 12 пациентов, в основном с макроАГ. ИГХ проведено 24 пациентам, и в 23 случаях выявлена выраженная экспрессия к ТТГ; в одной АГ выявлены лишь единичные клетки, экспрессирующие ТТГ. В работе Yamada S. и соавт. [16] при обнаружении в опухоли единичных клеток, экспрессирующих ТТГ, проводилась обработка протеиназой К для подтверждения диагноза; в нашем исследовании это не выполнялось, диагноз был подтвержден достижением биохимической ремиссии основного заболевания. При проведении логистического регрессионного анализа для диагностики предоперационных факторов, влияющих на ремиссию заболевания, мы выявили, что увеличение размера АГ на 1 мм повышает вероятность рецидива/отсутствия ремиссии в 1,15 раза, что соответствует результатам, полученным в предыдущих исследованиях [4][5][16][20]. Однако мы не получили статистически значимых результатов по параметру «инвазия в ходе операции», но, учитывая тенденцию критерия р, при расширении группы пациентов возможно достичь значимых результатов. Такие показатели, как ТТГ, свТ4, свТ3, ИФР-1, нарушение зрения, пол, возраст, не влияли на исход операции.

В связи с редкостью заболевания, разнообразием применяемых методов диагностики ремиссии, четких критериев излечения пациентов с ТТГ-АГ не установлено. Критерии полной ремиссии ТТГ-АГ включают: отсутствие клинической картины тиреотоксикоза, неврологической симптоматики, опухоли по данным визуализирующих исследований и нормализацию уровней ТТГ, свТ4 и свТ3 в сыворотке крови в течение 3–6 месяцев после операции [9]. В отличие от соматотропином, точный порог значения послеоперационного ТТГ для определения ремиссии не обозначен. Известно, что неопределяемый уровень ТТГ (менее 0,01 мЕд/л) может служить хорошим индикатором радикальности удаления ТТГ-АГ [4][7][19]. Также некоторые авторы оценивают ремиссию по ответу на стимуляцию тиролиберином или пробу с трийодтиронином, которая является наиболее чувствительным и специфичным тестом для подтверждения полного удаления АГ [19][29]. Таким образом, единого стандарта критериев ремиссии после операции не существует, и указанные выше факторы следует учитывать комплексно.

В работе Kim SH и соавт. исследовали уровень ТТГ после хирургического вмешательства (2, 6, 12, 18, 24 часа после операции) для выявления пациентов с риском рецидива заболевания уже в раннем послеоперационном периоде. Авторами было выявлено, что ТТГ спустя 12 часов более 0,62 мкМЕ/мл лучше всего предсказывал риск рецидива заболевания [20]. Мы также рассчитали на нашей серии случаев отрезную точку послеоперационного ТТГ (взятого спустя трое суток после операции) для определения пациентов с возможностью рецидива заболевания. По результатам анализа пациенты с отсутствием долгосрочной ремиссии имели уровень ТТГ на первой неделе после оперативного вмешательства выше 0,391 мМЕ/л (SP=88,9%, SE=100%), что может в дальнейшем помочь врачам в выборе тактики ведения пациентов и устанавливать индивидуальные сроки динамического наблюдения.

Альтернативными методами лечения ТТГ-АГ являются лучевая терапия и/или медикаментозное лечение АС. Их следует рассматривать при невозможности проведения оперативного вмешательства, неудачном хирургическом лечении или в случае отказа от операции.

Большинство тиреотропных клеток экспрессируют различное количество SSTR, особенно SSTR 2 и SSTR 5. Согласно опубликованным данным, АС эффективны примерно в 90–95% случаев и способствуют уменьшению размеров опухоли у 30–50% пациентов [1][30]. АС относительно безопасны, даже несмотря на побочные эффекты (распространенность 10–30%), такие как желчнокаменная болезнь, нарушение углеводного обмена, диарея. Дофаминовые рецепторы также представлены на тиреотропных клетках, поэтому АДР также могут применяться при некоторых ТТГ-АГ, но с невысокой эффективностью, положительные эффекты в основном наблюдаются у пациентов со смешанной секрецией ПРЛ/ТТГ-АГ [38].

Эффективность лучевой терапии составляет примерно 37% [39]. Однако, в связи с все большей доступностью АС, риском развития пангипопитуитаризма, данный вид терапии в настоящее время применяется значительно реже. Тем не менее лучевая терапия относительно более распространена при низкодифференцированных опухолях линии Pit-1, а также подходит для пациентов с противопоказаниями к хирургическому вмешательству и лекарственным препаратам, и пациентам с остаточной опухолевой тканью [22].

В нашем исследовании 4 пациентам с отсутствием ремиссии/рецидивом после первичного оперативного вмешательства были назначены АС длительного действия, и у всех достигнута ремиссия, что объясняется экспрессией SSTR2, по данным ИГХ. Из 9 неоперированных пациентов при применении АС длительного действия у 3 достигнута ремиссия заболевания, а также отмечено уменьшение размеров АГ на 30%. Лучевая терапия проведена всего в 2 случаях, и в обоих случаях ремиссия достигнута только после назначения АС. Назначение монотерапии АДР, а также в случае добавления их к уже имеющейся терапии, не приводило к стабилизации заболевания. В результате можно судить, что назначение АС длительного действия эффективно при комбинированном лечении пациентов с ТТГ-АГ.

Таким образом, схема диагностики и ведения пациентов с ТТГ-АГ может выглядеть так (рис. 4):

**Figure:**
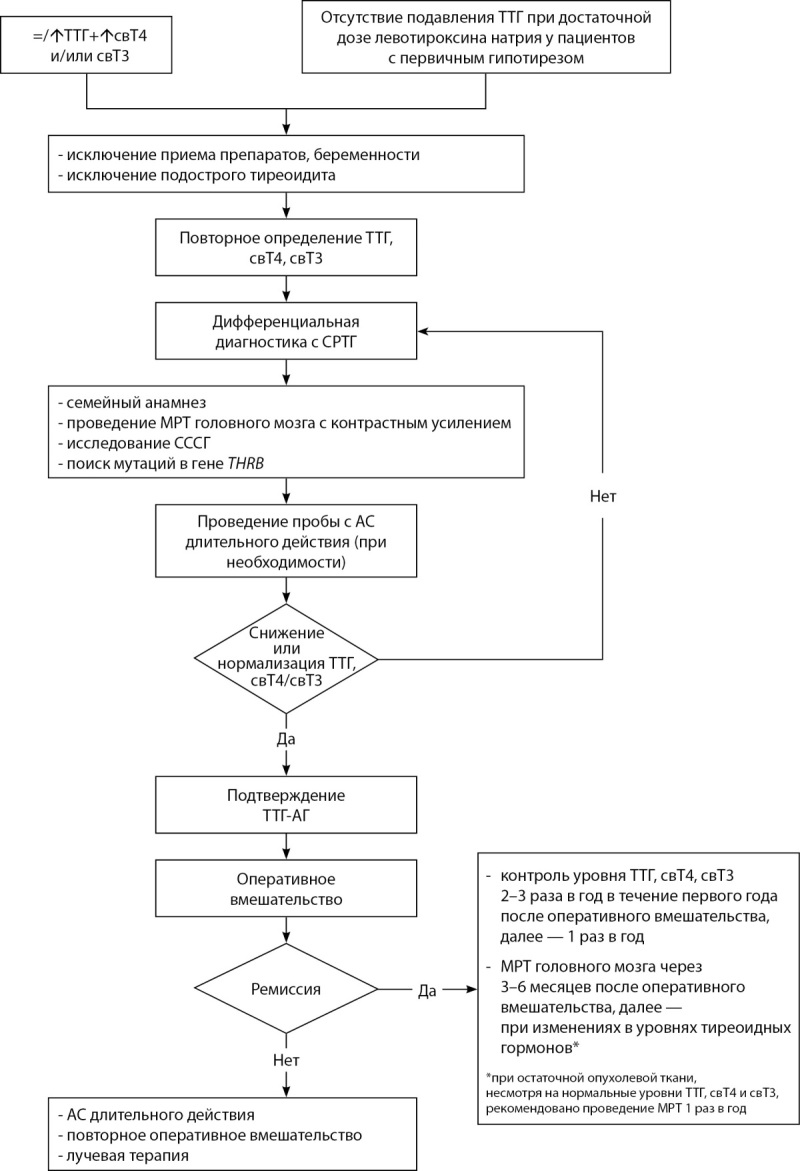
Рисунок. 4. Схема диагностики и ведения пациентов с ТТГ-АГ. Figure 4. Diagnostic and management scheme for patients with TSH-AH Примечание: ТТГ — тиреотропный гормон; свТ4 — тироксин; свТ3 — трийодтиронин; СРТГ — синдром резистентности к тиреоидным гормонам; THRB — ген рецептора тиреоидных гормонов бета; МРТ — магнитно-резонансная томография; СССГ — секс-стероид-связывающий глобулин; АС — аналог соматостатина.

## Ограничения исследования

Из-за ретроспективного дизайна исследования имеются некоторые ограничения анализа, в том числе отсутствие структурированного и последовательного наблюдения за пациентами, изменения в методах лечения с течением времени, неполные данные по определенным параметрам (например, ИГХ), потеря пациентов из-под наблюдения; а также ограничением является недоступность некоторых методов диагностики ТТГ-АГ.

## ЗАКЛЮЧЕНИЕ

ТТГ-АГ в структуре АГ встречаются крайне редко, а также являются достаточно редкой причиной тиреотоксикоза, вследствие чего, в большинстве своем, диагностируются на стадии макроаденомы. Крайне важную роль играют ранняя диагностика этих опухолей и дифференциальный диагноз ввиду часто неверного лечения пациентов с ТТГ-АГ. К сожалению, ни один из доступных методов в Российской Федерации (уровни тиреоидных гормонов, МРТ головного мозга, оценка СССГ, СТх, тест с октреотидом) не является идеальным для подтверждения диагноза ТТГ-АГ, поэтому их необходимо рассматривать комплексно. Наиболее эффективным методом лечения является трансназальная трансфеноидальная аденомэктомия (достижение ремиссии в 86,1%). В качестве терапии второй линии при неэффективности оперативного лечения, противопоказаниях или отказе пациента от оперативного вмешательства для контроля заболевания могут использоваться аналоги соматостатина (100% ремиссии при комбинированном лечении и 42,9% при монотерапии). Большие размеры АГ и инвазия в ходе операции могут быть в целом ассоциированы с неблагоприятным исходом хирургического лечения. Также нами предложена возможная модель уровня послеоперационного ТТГ (>0,391 мЕд/л) для прогнозирования рецидивов ТТГ-АГ, что требует подтверждения на большей группе пациентов.

## ДОПОЛНИТЕЛЬНАЯ ИНФОРМАЦИЯ

Источник финансирования. Работа выполнена в рамках гранта Министерства образования и науки, соглашение № 075-15-2022-310 от 20.05.2022.

Конфликт интересов. Авторы декларируют отсутствие явных и потенциальных конфликтов интересов, связанных с проведенным исследованием и публикацией настоящей статьи.

Вклад авторов. Все авторы подтверждают соответствие своего авторства международным критериям ICMJE (все авторы внесли существенный вклад в разработку концепции, проведение исследования и подготовку статьи, прочли и одобрили финальную версию перед публикацией).
